# Tuberculin skin test repetition after TNF-α inhibitors in patients with chronic inflammatory arthritis: a long-term retrospective cohort in endemic area

**DOI:** 10.1186/s42358-024-00406-7

**Published:** 2024-09-13

**Authors:** Vanessa de Oliveira Magalhães, Karina Rossi Bonfiglioli, Carina More Frade Gomes, Eloisa Bonfá, Ana Cristina de Medeiros-Ribeiro, Carla Gonçalves S. Saad, Marcelo de Medeiros Pinheiro

**Affiliations:** 1 Spondyloarthritis Section, Rheumatology Division, Federal University of São Paulo, (Unifesp/ EPM), Borges Lagoa Street, 913/ 51-53, Vila Clementino, São Paulo, SP 04038-034 Brazil; 2 Rheumatology DivisionHospital das Clínicas, Faculdade de Medicina (HCFMUSP), Universidade de São Paulo, São Paulo, Brazil; 3 Rheumatology Division, Federal University of Uberlândia (UFU), Uberlândia, MG Brazil

**Keywords:** Tuberculin skin test, TST, Tuberculosis, Latent tuberculosis infection, Chronic inflammatory arthritis, Rheumatoid arthritis, Ankylosing spondylitis, Psoriatic arthritis, TST repetition

## Abstract

**Objectives:**

To evaluate the tuberculin skin test (TST) conversion in chronic inflammatory arthropathies (CIA) patients on TNFα inhibitors (TNFi) and without previous latent tuberculosis infection (LTBI) treatment.

**Methods:**

Patients with rheumatoid arthritis (RA), ankylosing spondylitis (AS) and psoriatic arthritis (PsA) with negative LTBI were retrospectively evaluated for TST conversion and active tuberculosis (TB) after six months of exposition to TNFi. Two groups were compared: patients who repeated TST (*TST-repetition*) during the follow-up and patients who did not (*non-TST-repetition*).

**Results:**

A total of 355 CIA patients on TNFi were screened and 138 (38.9%) did not fulfill the inclusion criteria. Of the remaining 217 CIA patients, 81 (37.3%) repeated TST during TNFi treatment. TST conversion rate was observed in 18 (22.2%) patients without significant differences among CIA (*p* = 0.578). The number of TB cases was low (*n* = 10; 4.6%) and was similar in *TST-repetition* and *non-TST-repetition* groups [2 (2.5%) vs. 8 (5.9%), *p* = 0.328]. Of note, 30% of active TB occurred early (6–12 months of TNFi exposure) and the median (full range) time to incident TB was 1.3 (0.6–10.6) years, whereas the median (full range) time to TST repetition was later [3.3 (0.5–13.4) years]. The incidence of active TB was lower among RA patients than AS patients [342 (95% CI 41 − 1446) vs. 1.454 (95% CI 594-2993)/100,000 patient-years, *p* = 0.049].

**Conclusion:**

These results indicate that TST repetition is associated with a high conversion rate, suggesting the need for recommended treatment. The delayed repetition of TST and low number of active TB cases hampered the evaluation of this strategy effectiveness to prevent active infection. Larger studies with systematic repetition patterns are necessary. In addition, the study highlights the need for a greater surveillance for TB in AS patients.

## Key messages

• TST repetition is associated with a high conversion rate for positive LTBI after long-term TNFi.

• Active tuberculosis diagnosis occurs early (1.3 years) during TNFi therapy.

• AS patients require greater surveillance for incident TB.

## Introduction

Tuberculosis (TB) is an infectious and transmissible disease of great worldwide relevance caused by *Mycobacterium tuberculosis.* It is one of the ten leading causes of death worldwide and the most prominent related to a single infectious agent. According to the WHO (World Health Organization) data, about 10 million new cases and 1.5 million deaths are reported annually [1]. Brazil is one of the 30 countries with the highest prevalence in the world, with an estimated annual incidence of 35/100,000 inhabitants and mortality of 2.2/100,000 inhabitants [1, 2].

It is estimated that 5–10% of individuals with latent tuberculosis infection (LTBI) will develop tuberculosis throughout their lives, with a higher probability in populations at risk, such as people living with HIV, diabetes, malnutrition, alcoholics, individuals deprived of their liberty, drug addicted and patients under immunosuppressants, particularly TNFα inhibitors (TNFi) [3].

The main mechanism involved in TNFi higher TB risk is related to the granuloma disorganization after TNFα blockade or with re-exposure over time [4]. TB incidence in Brazilian patients with chronic inflammatory arthritis (CIA) using TNFi is high [5–6]. According to the multicenter register of Rheumatology Brazilian Society, the incidence among patients with RA under TNFi is 287/100,000 patient-years in contrast to 101/100,000 patient-years among RA patients under csDMARD) [5]. In South America, the overall incidence among CIA patients using TNFi is 11,750 cases/100,000 patient-years, greater than the incidence reported in such populations in the northern hemisphere (962 cases/100,000 patient-years) [6].

Therefore, LTBI identification is crucial to minimize the occurrence of tuberculosis during TNFi treatment [7–16]. However, some cases of tuberculosis have been reported even after screening strategies, especially in those patients with a negative LTBI investigation. Some of the non-reactive tests may be false negatives, probably due to anergy and immunosuppression status. This finding emphasizes the need of other tools that could be used to increase the sensitivity and specificity to identify more cases of LTBI, such as Interferon Gamma Release Assays (IGRAs) [17] and systematic TST repetition during long-term TNFi exposure as proposed by CDC (Center for Disease Control and Prevention) [13], WHO [12], ACR (American College of Rheumatology) and EULAR (European Alliance of Associations for Rheumatology) [15]. Although it has been demonstrated heterogeneous TST conversion rate (1.45–32.6%) [8, 18–23], after TNFi exposure in CIA patients with negative LTBI screening, it is worthy highlighting that is still unknown the ideal timing for repeating or whether it is associated with higher risk of active tuberculosis [15].

Hence, our objective was to assess tuberculin skin test (TST) conversion among patients with chronic inflammatory arthropathies (CIA) receiving TNFα inhibitors (TNFi) for at least 6 months and with no previous latent tuberculosis infection (LTBI) treatment. We also assessed active TB incidence in patients who repeated TST compared with those who did not.

## Patients and methods

### Patients

**Fig. 1:**
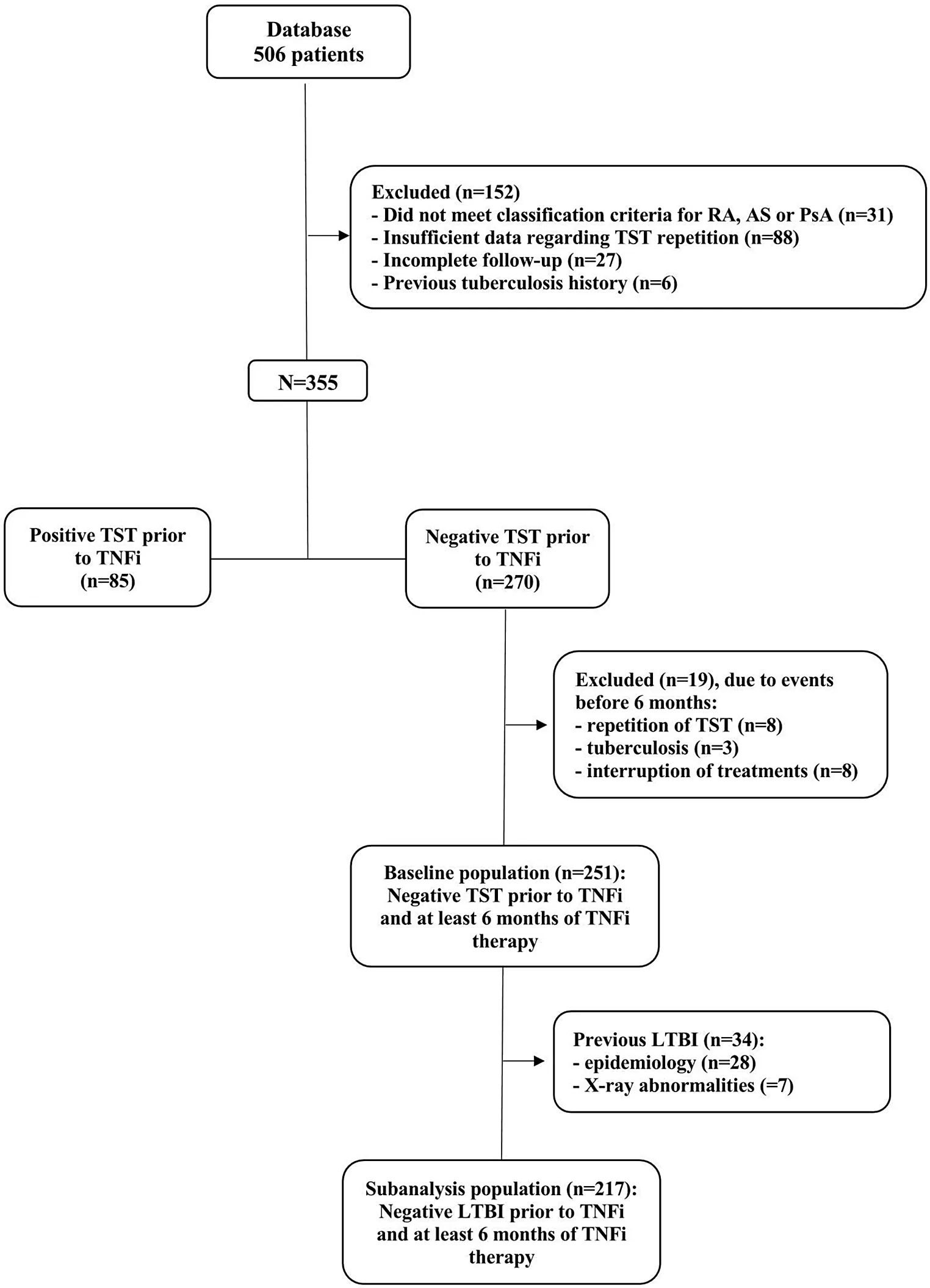
Flowchart of chronic inflammatory arthritis (CIA) patients undergoing TNFi therapy. *CIA* chronic inflammatory arthritis, *RA* rheumatoid arthritis, *AS* ankylosing spondylitis, *PsA* psoriatic arthritis, *TST* tuberculin skin test, *TNFi* tumor necrosis factor inhibitors, *LTBI* latent tuberculosis

This is a long-term, bicentric, retrospective cohort study including adult patients (*≥* 18 years old) with CIA, regularly followed at the Rheumatology Outpatient Clinics and Biological Centers of two public healthcare tertiary centers in Brazil. The centers have their own electronic medical records of all patients undergoing treatment with bDMARDs, including TNF inhibitors. The eligibility criteria for all patients under TNFi was fulfillment of the classification criteria for one of the following diseases: rheumatoid arthritis (RA) [24, 25], psoriatic arthritis (PsA) [26] and AS [27]. Patients should have received treatment with at least one TNFi (Etanercept, Adalimumab, Infliximab, Certolizumab Pegol, Golimumab) for at least 6 months, with a negative TST at baseline, and have not receive LTBI treatment for any other indication (X-ray or abnormalities or previous contact with TB) from June 1st, 2001, to December 31st, 2019 (Fig. 1). These patients were followed in the Biological Center of each Institution using an electronic chart database including all parameters relevant to this study (diagnosis, clinical evaluation at 1–3 months interval, or whenever necessary).

Exclusion criteria were: (1) Patients with other diagnosis than RA, AS or APS or not fulfilling the classification criteria for such diseases [24–27]; (2) Pulse therapy with cyclophosphamide and/or methylprednisolone in the last 12 months; (3) Severe comorbidities that could interfere with the outcomes (including neoplasia in the last five years, active HIV infection, hepatitis B and C, leprosy, sarcoidosis, pregnancy, lactation, infectious uveitis and demyelinating diseases); (4) Solid organ transplant or hematological disorders; (5) Chronic renal failure on dialysis; (6) Primary immunodeficiencies and thymus aplasia; (7) Positive TST prior to starting TNFi; (8) Patients with LTBI at baseline or who received previous treatment for LTBI or those with previously treated TB; (9) Patients without TST prior to starting TNFi treatment; (10) TST repetition due to suspicion of active tuberculosis during TNFi treatment; (11) Active TB diagnosis before 6 months of TNFi treatment (Fig. 1).

The final sample was divided in two groups: patients who repeated TST during the follow-up for re-screening purposes (*TST-repetition* group) and patients who did not (*non-TST-repetition* groups). In this study we evaluated only patients that repeated TST only once. Follow-up after any additional repetition was not analyzed.

### Outcomes

The primary outcome was the conversion rate of TST in the *TST-repetition* group. Secondary outcomes were frequency and incidence of active TB diagnosis during TNFi treatment.

### Methods

TST methodology consisted of inoculation of 0.1 ml (2UT) of PPD RT-23, intradermally in the forearm. After 48–72 h the induration area was measured by a trained professional and the maximum transverse diameter was expressed in millimeters [3, 12]. A positive test was considered if TST ≥ 5 mm [7, 11–14]. In case of positive TST, the patient received treatment for LTBI (isoniazid 300 mg per day for 6 months, according to the recommendations) [12].

The main reasons to repeat TST were administrative issues, including one of the mandatory items of the checklist for dispensing TNFi by local guidelines or due to loss of follow-up and need to restart the bureaucratic process according to the Brazilian Ministry of Health’s recommendations. The TST conversion rate was defined as the change from negative (0–4 mm) to positive (≥ 5 mm) after exposure to TNFi.

#### Tuberculosis

##### Latent tuberculosis infection (LTBI)

Prior to starting TNFi, a LTBI investigation was performed in all patients, including epidemiology assessment, X-ray chest imaging and TST. The same diagnostic approach was done by both the University Centers, except for the definition of epidemiology: patients from the Hospital das Clínicas [11] were considered as positive if any contact with tuberculosis was present throughout life and for those from the UNIFESP/EPM, only personal or familiar contact occurring in last two years was used [8]. All patients diagnosed with LTBI were treated and the TNFi initiation was postponed by a month, according to the established recommendations [9, 12]. Patients with negative TST were followed with close clinical surveillance for TB.

IGRA was not performed in this study due to unavailability.

##### Incident and active disease

Suspicious cases of TB at baseline and during follow-up were extensively evaluated with TST, imaging, and any other procedure necessary for diagnosis (sputum or tissue analyses, culture, or rapid molecular test for tuberculosis, as appropriate). Incident cases of active tuberculosis were defined as those that occurred after 6 months of TNFi therapy. Patients who developed active tuberculosis and performed a new TST to help the diagnosis, not as a screening for LTBI, were included in *non-TST-repetition* group. Sites were classified as pulmonary, extrapulmonary or miliary, according to the main affected organ.

#### Medications

For glucocorticoid (GC), a cut-off of stable dosage above 7.5 mg of prednisone/day or equivalent for at least 6 weeks prior to TST was established for the analysis of a possible deleterious effect of this drug taking into consideration that this dose was reported to result in supraphysiological concentrations [28]. Regarding the conventional synthetic DMARDs (drug-modifying antirheumatic drugs), such as methotrexate (MTX), leflunomide, sulfasalazine and azathioprine, in any dosage, for at least six weeks before TST was defined for the analysis of their potential harmful effect. The cut-off for MTX dosage was established as above 7.5 mg/week (orally or subcutaneously) whereas for leflunomide, sulfasalazine, and azathioprine categorization was dichotomous variables as either “yes” or “no”, regardless of dosage. For the biologic DMARDs (bDMARDs), an extensive analysis was performed concerning type (monoclonal and fusion protein), switching and clinical management after active TB (withdrawal; restarting; which agent; when; switch to another mechanism of action; disease activity and permanent discontinuation). Only patients on TNF inhibitors were included and followed up. Other mechanisms, such as JAK inhibitors and non-TNF biologic DMARDs, were excluded.

#### Ethic issues

The study was approved by the Ethics Committee of Research at the Federal University of Sao Paulo (CAAE:95120618.6.0000.5505) and at Hospital das Clinicas—University of Sao Paulo (CAAE:01795518.6.0000.0068), in accordance with the resolution 466 from CONEP (National Committee of Ethics in Research; CNS/466), as well as following all regulatory guidelines and standards regarding the data confidentiality and privacy, and the General Data Protection Law.

#### Funding statement

The authors received no financial support for the research, authorship, and/or publication of this article.

### Statistical analysis

Data were presented through descriptive analysis, including absolute (n) and relative (%) frequency distribution, as well as median and full ranges. The inferential analysis used the chi-square association test or Fisher’s exact test to assess the relationship between numerical variables, as appropriate. The comparison of continuous variables in relation to the groups of patients with CIA was performed using the non-parametric Kruskal-Wallis one-way analysis of variance on ranks. In the case of a significant difference, the Bonferroni correction multiple comparison test was performed to identify which pairs of groups differed from each other. Unadjusted logistic regression analyses were performed considering incident tuberculosis as a dependent variable. The adjusted logistic regression models were performed with incident tuberculosis as dependent variable and all other as independent ones when *p* < 0.20 in each univariate analysis.

Active tuberculosis incident-density data (with 95% confidence intervals) were estimated using Poisson distribution and test-based methods and compared among pairs of CIA or diseases. The incident-density was calculated using the time of exposure to TNFi after TST repetition in those who repeated TST. Calculation of the incidence rate during TNFi therapy was performed as the ratio between the number of cases observed divided by the patients-years of follow-up and converted for the ratio per 100,000 patient-years to better compare with previous published data. To calculate the incident-density after the first screening, the exposure time to TNFi was the sum of time 6 months after starting TNFi in those who did not repeat TST plus the time 6 months after TNFi treatment up to TST repetition date of those who repeated TST.

## Results

A total of 506 CIA patients were enrolled in this retrospective cohort study and 151 (29.6%) were excluded due to insufficient data regarding TST repetition, and other ineligibility criteria, such as previous tuberculosis history, or incomplete follow-up. Among the remaining 355 patients, 85 (23.9%) had positive and 270 (76.1%) had negative TST before starting TNFi at baseline. Among the baseline TST negative patients, those with LTBI at baseline (*n* = 34) due to epidemiology (*n* = 28) and/or X-ray abnormalities (*n* = 7) were also excluded. Nineteen patients were additionally excluded due to repetition of TST (*n* = 8), active tuberculosis (*n* = 3) or other causes to TNFi withdrawal (*n* = 8), all before 6 months. Two patients with axial spondyloarthritis and 7 with rheumatoid arthritis had a history of previously treated tuberculosis.

The study population comprised 217 patients with baseline negative LTBI, of whom 111 (51.1%) with RA, 77 (35.5%) with AS and 29 (13.4%) with PsA (Fig. 1). Patient’s age (*p* < 0.001) and gender (*p* < 0.001) were distinct among groups and within the expected distribution for each disease, likewise the association to glucocorticoids and conventional DMARDs. TNF exposure time (*p* = 0.125) was comparable among the 3 CIA groups. Switchers were more frequent in the RA group in comparison to AS patients (*p* < 0.001) (Table 1).

**Table 1:** Baseline clinical and demographic characteristics of negative tuberculin skin test (TST) chronic inflammatory arthritis (CIA) patients who survived the first 6 months of TNFi therapy

	All patients *n* = 217	RA (*n* = 111) (51.1%)	AS (*n* = 77) (35.5%)	PsA (*n* = 29) (13.4%)	*P*
Age, years	47.7 (14.8–83.3)	50.6 (21.2–80.6)	39.7 (14.8–71.8)	49.3 (19.7–83.3)	< 0.001^a,c^
Men, n (%)	88 (40.6)	13 (11.7)	61 (79.2)	14 (48.3)	< 0.001^a,b,c^
Disease duration, years	9 (0–36)	10 (0–34)	7 (0–36)	9 (0–30)	0.036^d^
RF, n (%)	NA	90 (81.1)	NA	NA	–
HLA-B27, n (%)	NA	NA	44/52(84.6)	2/4(50)	0.142
Treatment					
TNFi*					
ADA, n (%)	127 (58.5)	62 (55.9)	46 (59.7)	19 (65.5)	0.620
ETA, n (%)	94 (43.3)	57 (51.4)	27 (35.1)	10 (34.5)	0.050
IFX, n (%)	129 (59.4)	70 (63.1)	42 (54.5)	17 (58.6)	0.502
CZP, n (%)	8 (3.7)	2 (1.8)	5 (6.5)	1 (3.4)	0.244
GOL, n (%)	8 (3.7)	1 (0.9)	4 (5.2)	3 (10.3)	0.038^b^
TNFi Switchers, n (%)	118 (54.4)	73 (65.8)	30 (39)	15 (51.7)	0.001^a^
Time of TNFi exposure (years)	6.0 (0.5–17)	5.5 (0.5–17)	6.9 (0.5–14.1)	6.8 (0.6–13.1)	0.125
GC	88 (40.6)	75 (67.6)	8 (10.4)	5 (17.2)	< 0.001^a,b^
Any csDMARD, n (%)	170 (78.3)	104 (93.7)	42 (54.5)	24 (82.8)	< 0.001^a,c^
MTX, n (%)	142 (65.4)	93 (83.8)	28 (36.4)	21 (72.4)	< 0.001^a,c^
LEF, n (%)	80 (36.9)	67 (60.4)	5 (6.5)	8 (27.6)	< 0.001^a,b,c^
SSZ, n (%)	68 (31.3)	36 (32.4)	30 (39)	2 (6.9)^a,b^	0.006^b,c^
AZA, n (%)	22 (10.1)	19 (17.1)	1 (1.3)	2 (6.9)	0.002^a^

The results are expressed in number (%) or in median and full range (minimum-maximum interval)

*NA* not available, *RA* rheumatoid arthritis, *AS* ankylosing spondylitis, *PsA* psoriatic arthritis, *RF* rheumatoid factor, *TB* tuberculosis, *TNFi* tumor necrosis factor inhibitor, *IFX* infliximab, *ETA* etanercept, *ADA* adalimumab, *CZP* certolizumab Pegol, *GOL* golimumab, *GC* glucocorticosteroids, *csDMARD* conventional synthetic disease modifying anti-rheumatic drugs, *MTX* methotrexate, *LEF* leflunomide, *SSZ* sulfasalazine, *AZA* azathioprine

*Cumulative rate of TNFi’s prescriptions, regardless of multiple switching

^a^significant difference (*p* < 0.05) between RA and AS

^b^significant difference between RA and PsA

^c^significant difference between AS and PsA

^d^no specific difference among groups after correction

More than one third of the patients (*n* = 81; 37.3%) repeated TST, without differences among the CIA (*p* = 0.794). Repetition was performed after a median (full range) of 3.3 (0.5–13.4) years on TNFi. TST conversion rate was observed in 18 (22.2%) patients, with no differences among CIA (*p* = 0.578) (Table 2). These patients received adequate LTBI treatment, with no major side effects or drug discontinuation.

**Table 2:** TST repetition and conversion rates, and active tuberculosis frequency and incidence after exposure to TNF inhibitors in patients with chronic inflammatory arthritis without baseline LTBI (negative baseline TST, no X-ray abnormalities, no previous history of contact)

	All CIA patients (*n* = 217)	RA (*n* = 111) (51.1%)	AS (*n* = 77) (35.5%)	PsA (*n* = 29) (13.4%)	
Repetition TST, n (%)	81 (37.3)	40 (36.0)	31 (40.3)	10 (34.5)	0.794
Median (full range) time of TST repetition, years	3.3 (0.5–13.4)	2.6 (0.5–11.3)	3.7 (0.7–13.4)	3.5 (0.9–7.7)	0.046^a^
Conversion to TST ≥ 5 mm, n (%)	18 (22.2)	7 (17.5)	8 (25.8)	3 (30)	0.578
TB diagnosis, n (%)	10 (4.6)	2 (1.8)	7 (9.1)	1 (3.4)	0.061
Median (full range) time to TB diagnosis, years^a^	1.3 (0.6–10.6)	1.1 (0.7–1.4)	1.8 (0.6–10.6)	1.3 (1.3–1.3)	0.386
TB between 6 and 12 months of TNFi therapy, n (%)	3 (30)	1 (50)	2 (28.6)	0 (0)	0.665
**Active TB during TNFi therapy**					
*Non-TST-repetition* group, n (%)	8 (5.9)	2 (2.8)	5 (10.9)	1 (5.3)	0.194
*TST-repetition* group, n (%)	2 (2.5)	0	2 (6.5)	0	0.191
*P*	0.328	0.535	0.695	> 0.999	
**Incidence rate of active TB during TNFi therapy/100,000 patient-years**					
Overall	797 (382–01446)	342 (41–1234)	1454 (594–2993)	535 (14–2980)	0.049^a^
Number of incident events/patient-years followed	10 / 1254.2	2 / 585.4	7 / 481.8	1 / 187	
After the 1st screening (without/before TST repetition)	941 (406–1855)	510 (62–1843)	1502 (488–3506)	800 (20–4457)	0.176^a^
Number of incident events/patient-years followed	8 / 849.9	2 / 392.1	5 / 332.8	1 / 125	
After TST repetition	495 (60–1787)	0 (0–1910)	1342 (163–4849)	0 (0–5950)	0.107^a^
Number of incident events/patient-years followed	2 / 404.3	0 / 193.3	2 / 149	0 / 62	
*p*	0.408	0.321	0.893	0.481	

The results are expressed in number (%) or in median and full range (minimum-maximum interval). Incidence rates are expressed as rates per 100,000 patients-years (95% confidence interval). The number of incident events (active tuberculosis during treatment)/the number of patient-years followed is depicted under each incidence rate

*CIA* chronic inflammatory arthritis, *RA* rheumatoid arthritis, *AS* ankylosing spondylitis, *PsA* psoriatic arthritis, *TB* tuberculosis, *TNFi* tumor necrosis factor inhibitors, *TST* tuberculin skin test

^a^significant difference (*P* < 0.05) between RA and AS patients

The comparison between TST-repetition and non-TST-repetition (Table 3) groups showed that (Table 3) groups, a higher frequency of etanercept use was noted in the TST-repetition group (*p* < 0.001). Conversely, a higher use of monoclonal TNFi antibodies was noted in the non-TST-repetition group (*p* < 0.001). An in-depth analysis showed that frequency of switchers were similar between both groups, without differences regarding the pattern of choice of the second TNFi (11.1% [*n* = 5/45] vs. 5.5% [*n* = 4/73], *p* = 0.299). Among non-switchers, etanercept users also presented a higher frequency of TST repetition: 41.7% (*n* = 15/36) vs. 11.1% (*n* = 7/63) (*p* < 0.001).

**Table 3:** Comparison of the clinical and demographic characteristics at baseline between the TST repetition group and Non-TST repetition group during follow up

	TST repetition group (*n* = 81)	Non-TST repetition group (*n* = 136)	*p*
Age (years)	47.4 (14.8–67.6)	47.9 (18.2–83.3)	0.905
Male gender, n (%)	32 (39.5)	56 (41.2)	0.886
RA, n (%)	40 (49.4)	71 (52.2)	0.779
AS, n (%)	31 (38.3)	46 (33.8)	0.558
PsA, n (%)	10 (12.3)	19 (14.0)	0.837
Previous/current treatment			
Sulfasalazine, n (%)	23 (28.4)	45 (33.1)	0.545
Methotrexate, n (%)	53 (65.4)	89 (65.4)	> 0.999
Azathioprine, n (%)	4 (4.9)	18 (13.2)	0.063
Leflunomide, n (%)	29 (35.8)	50 (36.8)	> 0.999
Prednisone, n (%)	30 (37.0)	57 (41.9)	0.479
Etanercept, n (%)	52 (64.2)	46 (33.8)	< 0.001
MAB, n (%)	61 (75.3)	128 (94.1)	< 0.001
Adalimumab, n (%)	43 (53.1)	83 (61.0)	0.259
Infliximab, n (%)	42 (51.9)	87 (64.0)	0.079
Certolizumab, n (%)	1 (1.2)	7 (5.1)	0.263
Golimumab, n (%)	4 (4.9)	4 (2.9)	0.475
Switchers, n (%)	45 (55.6)	73 (53.7)	0.252

The results are expressed in number (%) or in median and full range (minimum-maximum interval)

*RA* rheumatoid arthritis, *AS* ankylosing spondylitis, *PsA* psoriatic arthritis, *MAB* monoclonal antibody

During the follow-up of patients who survived the first 6 months of TNFi treatment, there were 10 (4.6%) new cases of active TB (Table 2). The overall incidence of TB was 797/100,000 (95% CI 382-1446) patient-years (Table 2). The incidence was lower among RA patients in comparison to AS patients (*p* = 0.049) and lower in the TST-repetition group in comparison to the *non-TST-repetition* group, but not reaching statistical significance (Table 2).

Three (30%) active TB cases were diagnosed between 6 and 12 months of treatment, similarly among CIA groups (*p* = 0.665). The median time elapsed from TNFi initiation and TB onset was 1.3 (0.6–10.6) year and was also comparable among CIA (*p* = 0.386) (Table 2). All patients had pleuropulmonar TB. The most frequently used TNFi at TB diagnosis were infliximab (IFX) (*n* = 5; 50%) and adalimumab (ADA) (*n* = 4; 40%), followed by etanercept (ETA) (*n* = 1; 10%) (Table 4). Five (50%) patients who developed active tuberculosis performed a new TST to help the diagnosis, which was positive in all these patients (100%).

**Table 4:** Active tuberculosis cases in chronic inflammatory arthritis (CIA) patients who survived the first 6 months of TNF inhibitors with negative LTBI at baseline

Patient	Dx	Age (years)	Sex	Baseline TST (mm)	Repeated TST	2nd TST (mm)	Time to TST repetition (years)	Time to TB after TNFi (years)	TNFi at TB diagnosis	Site of active TB
Cases among patients who did not repeat TST (*n* = 136)										
1	AS	53	M	0	No	–	–	0.6	IFX	Lung
2	RA	56	F	0	No	–	–	0.7	IFX	Lung
3	AS	59	M	3	No	–	–	1.0	ETA	Lung
4	AS	21	M	0	No	–	–	1.2	IFX	Lung
5	PsA	49	M	0	No	–	–	1.3	IFX	Lung
6	RA	56	F	4	No	–	–	1.4	IFX	Lung
7	AS	26	M	0	No	–	–	1.8	ADA	Lung
8	AS	45	M	0	No	–	–	3.5	ADA	Lung
Cases among patients with a second negative TST (*n* = 63)										
9	AS	32	F	0	Yes	0	3.4	7.1	ADA	Lung
Cases among patients who converted TST (*n* = 18)										
10	AS	35	M	0	Yes	8*	2.5	10.6	ADA	Lung

*Dx* diagnosis, *TST* tuberculin skin test, *TB* tuberculosis, *TNFi* tumor necrosis factor inhibitors, *AS* ankylosing spondylitis, *RA* rheumatoid arthritis, *PsA* psoriatic arthritis, *M* male, *F* female, *IFX* infliximab, *ETA* etanercept, *ADA* adalimumab

*This patient was properly treated for LTBI for 6 months by the occasion of the TST repetition and after exclusion of active tuberculosis

Two cases occurred in the *TST-repetition* group: one treated for LTBI based on TST conversion 2.5 years after initiating TNFi and developed active TB 8 years after the LTBI treatment; and the other was among the persistent negative retested patients (*n* = 63). The latter patient repeated TST 3.4 years after initiating TNFi and active tuberculosis occurred almost 4 years later (Table 4).

The unadjusted regression model showed that AS was associated with a higher risk of incident active tuberculosis [OR = 4.57; 95% CI 1.15–18.2, *p* = 0.031], while the use type of TNFi did not (*p* > 0.05). However, no significant risk factor was found after adjustments for confounders in the final regression model (Table 5).

**Table 5:** Unadjusted and adjusted logistic regression models examining the factors associated with active tuberculosis occurrence 6 months after initiating TNFi for chronic inflammatory arthritis with negative baseline LTBI (negative baseline TST, no history of contact and no X-ray abnormalities

Risk factors	Unadjusted OR (95% CI)	*P* value	Adjusted OR (95% CI)	*P* value
Age	0.96 (0.92–1.01)	0.137	0.98 (0.93–1.03)	0.392
Men, *n* = 88	3.63 (0.91–14.44)	0.067	1.79 (0.30–10.52)	0.520
RA, *n* = 111	0.22 (0.05–1.08)	0.063	0.64 (0.05–8.86)	0.739
AS, *n* = 77	4.57 (1.15–18.20)	0.031	1.69 (0.16–17.55)	0.662
PsA, *n* = 29	0.71 (0.09–5.82)	0.750		
Repetition TST, *n* = 81	0.41 (0.08–1.96)	0.261		
Positive Repetition TST, *n* = 18/81	3.65 (0.22–61.39)	0.369		
Prednisone, *n* = 88	0.62 (0.15–2.45)	0.490		
ADA, *n* = 127	1.07 (0.29–3.89)	0.923		
ETA, *n* = 94	1.33 (0.37–4.72)	0.663		
IFX, *n* = 129	2.84 (0.59–13.72)	0.193	3.31 (0.66–16.43)	0.144
CZP, *n* = 8	–	0.998		
GOL, *n* = 8	–	0.998		
MAb, *n* = 194	1.07 (0.13–8.85)	0.950		
TNFi switchers, *n* = 118	1.27 (0.35–4.64)	0.715		
Any csDMARD, *n* = 170	0.39 (0.11–1.46)	0.162	0.60 (0.13–2.74)	0.513
MTX, *n* = 142	0.51 (0.14–1.82)	0.301		
LEF, *n* = 80	0.41 (0.08–1.98)	0.268		
SSZ, *n* = 68	2.29 (0.64–8.18)	0.204		
AZA, *n* = 22	–	0.998		

Adjusted analyses included the factors with *p* > 0.20 at unadjusted analyses

*OR* odds ratios, *CI* confidence interval, *RA* rheumatoid arthritis, *AS* ankylosing spondylitis, *PsA* psoriatic arthritis, *LTBI* latent tuberculosis infection, *TNFi* tumor necrosis factor inhibitor, *TST* tuberculin skin test, *ADA* adalimumab, *ETA* etanercept, *IFX* infliximab, *CZP* certolizumab Pegol, *GOL* golimumab, *MTX* methotrexate, *LEF* leflunomide, *SSZ* sulfasalazine, *AZA* azathioprine, *csDMARD* conventional synthetic disease modifying anti-rheumatic drugs

All patients who had a positive TST conversion during the study were treated for latent tuberculosis according to current recommendations. They were clinically assessed and monitored. None of them developed incident active tuberculosis during the follow up.

## Discussion

Our study provides additional data by focusing exclusively on LTBI-negative CIA patients that have been exposed to long-term TNFi therapy, revealing a significant rate of TST conversion. We confirmed a predominance of incident cases before 2 years, even after excluding TB cases before 6 months, which probably hampered the effectiveness of delayed TST repetition (median > 3 years).

The TST conversion rate after repetition over time observed herein was concordant with other cross-sectional studies, in which the conversion was between 2% and 32%, using the same cut-off of 5 mm for TST positivity after repetition [7, 18–23], with possible variations due to time of repetition and geographic variations in the TB prevalence.

The great advantage of this study is the long-term follow-up of CIA patients exposed to TNFi therapy in an endemic country and without LTBI at baseline, according to the well recommended screening strategy in Brazil, including TST, epidemiologic history and typical chest X-ray findings [1, 29]. The exclusion of patients with baseline LTBI, who already received LTBI treatment, was essential since the repetition of TST in this subgroup of patients is not recommended [30]. However, the IGRA test was not performed in this study, since it was unavailable in public health centers in Brazil up to 2023, and previous studies demonstrated that it could improve the LTBI screening performance, especially in TST-negative patients due to anergy [8, 31–34].

The reasons for this high TST conversion rate after exposure to TNFi could be directly related to re-exposure, improved cellular response after treatment with TNFi and reduction of GC dosage [28, 35]. The present study suggests that the possibility of a booster phenomenon is remote, as it does not meet the time interval of 6 weeks between the two tests, according to the current definition [35]. For AS patients, it may reflect a new contact with the bacillus, since these patients usually do not have an impairment of cellular response [8, 36].

On the other hand, anergy is widely demonstrated in RA [7, 11, 17, 21, 22, 36–39] and, less extensively, in PsA patients [7, 40] and may account for the observed negative baseline TST. The improvement of immunogenicity in RA patients is probably the major factor accounting for TST conversion, since the treatment with TNFi is associated with reduction of glucocorticoids dosage and better control of disease activity [40]. Ehrenstein et al. demonstrated changes in the number and function of regulatory T cells (CD24 + CD25+) in patients with active RA and, after the use of infliximab, a restoration of the regulatory capacity of these cells, as well as a quantitative increase in peripheral blood [33].

The present trial used incident TB as a secondary endpoint, but failed to show such a possible benefit since the repetition of TST was performed late during TNFi exposition. The overall TB incidence was 797/100,000 (95% CI 382-1446) patient-years, quite similar to a recent systematic review and meta-analysis that included 52 observational studies and 98,483 patients, which found a global incidence of 960/100,000 exposed patients and 11,750 cases/100,000 patient-years in South America [34]. The low number of cases precluded the distinction among the monoclonal antibodies and ETA, as previously described [5, 41, 42–43]. Moreover, the higher frequency of ETA prescription in the TST-repetition group, found both in switchers and non-switchers, is another important limitation since the occurrence of TB is lower among ETA users in comparison to MAb TNFi users.

We confirmed previous findings showing that AS patients presented a higher incidence of TB than RA patients [6], despite the lower risk of anergy in the former group [7, 11, 40]. We attributed such aspect to epidemiological reasons, since AS patients are more frequently men and younger, in a productive age, than RA patients. These two factors are associated with higher TB exposure and higher associated risk [44].

Regarding the time of occurrence of active TB during the long follow-up of this study, 30% of cases occurred before 12 months of TNFi use, pointing to the reactivation of TB as a possible contributing factor in such cases. Of note, Carmona *at al.* demonstrated the effectiveness and safety of strategies to investigate LTBI and prevent reactivation in the first years of TNFi treatment [9]. In contrast, cases that occurred lately were probably due to TB re-exposure, since the presentation of TB in all patients was pulmonary [5, 40], while very early cases of TB due to reactivation are commonly associated with extrapulmonary presentations [5, 40].

Of note, the higher frequency of AS patients under csDMARDs is probably related to the fact that Brazilian patients have more frequent peripheral involvement than Caucasian AS patients [44–47]. In fact, the prescription choice is in accordance with the recommendations outlined in the Brazilian guidelines and supported by other pertinent national literature [48]. Despite this pattern prescription, we have not observed any impact of corticosteroids, synthetic DMARD on TST conversion rate or occurrence of active TB over time, but longitudinal changes on use, dose, or time of exposure to synthetic DMARD or prednisone were not evaluated.

The study has some limitations, such as the retrospective design, the non-systematic and late repetition of TST. This design may create a survival bias since TST was repeated in a TB reactivation low-risk group of patients who survived to the first years of treatment. In fact, the observation of a low number of TB cases after two years of exposition, regardless of the repetition of TST, points that late expositions can occur but are uncommon, in accordance with previous described good long-term prognosis for patients who did not have early general infections during bDMARD first months [49]. Moreover, there was also a lack of power to evaluate effectiveness of TST repetition. In this context, it remains unclear if early systematic repetition of TST could help to identify previous or a new exposition and the need of LTBI treatment to prevent active TB cases Therefore, larger and systematic trials are necessary to define more accurately the best moment to repeat TST and its effectiveness. Another limitation is the inclusion of patients with greater severity and complexity, followed up in academic hospitals, with a profile of multiple and heterogeneous risk factors for TB [43]. In addition, we did not evaluate the IGRAs in this cohort because they were not widely available in Brazil until late 2023. Information regarding tobacco exposition, COPD, and detailed csDMARD prescription was not available.

In conclusion, our results indicate that TST repetition is associated with a high conversion rate, emphasizing the importance of implementing treatment according to existing guidelines. However, the assessment of this strategy’s effectiveness in preventing active TB was hampered by the delayed repetition and limited sample size of active TB cases. Larger studies incorporating systematic repetition patterns are imperative for a comprehensive evaluation. In addition, our study highlights the need for enhanced surveillance for incident TB in AS patients.

## Data Availability

The authors confirm that the data supporting the findings of this study are available within the article and its supplementary materials.
